# The role of unconventional T cells in COVID-19

**DOI:** 10.1007/s11845-021-02653-9

**Published:** 2021-05-29

**Authors:** Kristen Orumaa, Margaret R. Dunne

**Affiliations:** Department of Clinical Microbiology and Department of Immunology, Trinity Translational Medicine Institute, St James’s Hospital, Dublin 8, Ireland

**Keywords:** COVID-19, INKT cells, Γδ T cells, MAIT cells, SARS-CoV-2, Unconventional T cells

## Abstract

COVID-19 is a respiratory disease caused by the severe acute respiratory syndrome coronavirus 2 (SARS-CoV-2). It was first documented in late 2019, but within months, a worldwide pandemic was declared due to the easily transmissible nature of the virus. Research to date on the immune response to SARS-CoV-2 has focused largely on conventional B and T lymphocytes. This review examines the emerging role of unconventional T cell subsets, including γδ T cells, invariant natural killer T (iNKT) cells and mucosal associated invariant T (MAIT) cells in human SARS-CoV-2 infection.

Some of these T cell subsets have been shown to play protective roles in anti-viral immunity by suppressing viral replication and opsonising virions of SARS-CoV. Here, we explore whether unconventional T cells play a protective role in SARS-CoV-2 infection as well. Unconventional T cells are already under investigation as cell-based immunotherapies for cancer. We discuss the potential use of these cells as therapeutic agents in the COVID-19 setting. Due to the rapidly evolving situation presented by COVID-19, there is an urgent need to understand the pathogenesis of this disease and the mechanisms underlying its immune response. Through this, we may be able to better help those with severe cases and lower the mortality rate by devising more effective vaccines and novel treatment strategies.

## Introduction

SARS-CoV-2, a virus which causes the disease known as COVID-19, was first described in a case of pneumonia of unknown origin in Wuhan City, China [1] but quickly evolved into a worldwide pandemic. As of March 30th 2021, there have been 127,349,248 confirmed cases of SARS-CoV-2 with 2,787,593 confirmed deaths, and roughly, for every 46 confirmed cases, there is 1 confirmed death [2]. As such, there is an urgent need to stop the spread of SARS-CoV-2 and minimise related fatalities. However, this cannot be done without first understanding the pathogenesis of this novel disease. The immune system is thought to play a key role in COVID-19 pathogenesis, but to date, the majority of immune studies have focussed on conventional B and T cells. This review will discuss emerging data on the role of lesser-studied unconventional T cell subsets in the SARS-CoV-2 infection. Unconventional T cells, such as γδ T cells, iNKT cells and MAIT cells, have been implicated in host defence against microbes and cancer, mediating rapid and potent killing of infected or abnormal cells. Evaluating the response of such cells in COVID-19 will extend the breadth of knowledge regarding immune antiviral effector responses, and potentially highlight novel therapeutic targets.

## SARS-CoV-2

Clinicians determined that a cluster of cases of pneumonia in Wuhan was virus-induced which led to samples from seven patients being tested for coronavirus. The samples were tested for coronavirus because the environment of the market where these cases originated was similar to those where other SARS infections began. Of the seven samples tested, five were PCR-positive for coronavirus [3]. Analysis of amino acid sequences of seven conserved replicase domains showed that SARS-CoV and SARS-CoV-2 were 94.4% identical, suggesting that they belonged to the same virus family. The SARS-CoV-2 genome shares about 80% sequence identity with SARS-CoV and approximately 50% with Middle East Respiratory Syndrome Coronavirus (MERS-CoV) [3].

The most recent coronavirus infections were SARS-CoV in 2002 which was identified in Guangdong Province, China [4], and MERS-CoV in 2012 which was identified in Saudi Arabia [5]. As demonstrated in Table 1, SARS-CoV and MERS-CoV had fewer confirmed cases when compared with SARS-CoV-2; however, their fatality rate was higher, especially for MERS-CoV. Based on the data in Table 1, MERS-CoV is the most fatal coronavirus infection while SARS-CoV-2 is the most contagious of the three [2, 6–9].

**Table 1:** A comparison of the confirmed cases worldwide, casualties, fatality rate and reproductive number (R number) of SARS-CoV, MERS-CoV and SARS-CoV-2

Coronavirus	Confirmed cases	Casualties	Fatality rate	R number	Timeframe	Reference
SARS-CoV	8,422	916	10.87%	0.95	2002–2003 2004–2004	[7, 9]
MERS-CoV	2,581	935	36%	0.91	2012–Jan 2021	[7, 8]
SARS-CoV-2	127,349,248	2,787,593	2.2%	2.87	Dec 2019–Mar 2021	[2, 6]

## Clinical features of COVID-19 

The main mode of transmission for SARS-CoV-2 is through respiratory droplets with an incubation period ranging from 2 to 14 days [10]. However, 97.5% of individuals develop symptoms within 11–12 days [11]. Individuals with COVID-19 experience a wide range of clinical manifestations, but it is also possible to be COVID-19 positive and exhibit no symptoms [12]. Based on a WHO situation report from March, 2020, 80% of cases are mild/asymptomatic, 15% are severe (require oxygen) and 5% are critical (require ventilation) [13]. While asymptomatic patients do not present clinical symptoms, infection can be discovered based on abnormal lung findings on CT scans [14] or through PCR testing [15]. Interestingly, another study suggests that the infectivity of asymptomatic carriers is weak [16]. In the case of symptomatic patients, common symptoms of the illness include fever, cough and fatigue while severe cases are more commonly characterised by dyspnoea, lymphopenia and hypoalbuminemia [17]. COVID-19 has also been shown to have a male bias as, in comparison to females, male patients with COVID-19 are three times more likely to require intensive care unit (ICU) treatment and have higher odds of death [18].

The three most common symptoms in patients with mild COVID-19 are fever, cough and expectoration with the absence of complications such as acute respiratory distress syndrome (ARDS), acute respiratory system injury, acute kidney injury or septic shock [19]. Another study found hyposmia to be another common symptom and it was often accompanied by hypogeusia, nasal congestion or rhinorrhoea [20]. As well as this, the most prevalent comorbidities are hypertension, diabetes, cardiovascular disease and respiratory system disease [21].

COVID-19 has also been shown to have a clear age bias [22]. The possibility of a severe COVID-19 diagnosis increases with older age, lower lymphocyte count and pulmonary opacity in CT upon admission to hospital [22, 23]. Severe cases are characterised by extensive lung damage, lymphopenia, neutrophilia and macrophage and neutrophil infiltrates being observed in blood and lung tissues [24]. Moreover, older patients with comorbidities such as hypertension and diabetes are more likely to develop severe COVID-19 [23, 25], with underlying respiratory and cardiovascular disease hypothesised to contribute towards the severity also [21]. Furthermore, it has also been observed that older patients who died due to COVID-19 had higher incidences of hypertension, coronary disease and dyspnoea upon admission along with low blood gas index and poor coagulation [26].

## The immune response

The immune system is composed of the innate and adaptive arms. The innate immune system is activated after an invading pathogen is recognised by pattern recognition receptors, and once activated, it is responsible for the recruitment of immune cells to the site of inflammation or infection through the activation of chemokine and cytokine signalling cascades [27]. Of these chemokines and cytokines, the production of type I interferons (IFNs) and inflammatory cytokines are considered to be hallmarks of the antiviral immune response [28]. The cellular branch of the innate immune system includes macrophages/monocytes, neutrophils, natural killer (NK) cells and dendritic cells (DC) [29].

The activation of adaptive immunity is orchestrated by the innate immune system and this response is even more important if innate immunity has failed to contain or eliminate the infectious agent. The adaptive immune system consists of T and B lymphocytes that respond to “non-self” antigens. This facilitates pathogen-specific immunologic effector pathways. As well as this, immunologic memory is developed and host immune homeostasis must be maintained during this response [30]. T cell receptors (TCR) on T lymphocytes recognise antigens presented by antigen presenting cells (APCs). This results in rapid proliferation and differentiation of antigen-specific lymphocytes in a process known as clonal expansion. APCs present antigens to T cells in a complex with proteins known as major histocompatibility complex (MHC) with MHC class I molecules presenting antigens to CD8^+^ T cells and MHC class II molecules presenting to CD4^+^ T cells [27].

## The innate immune response to COVID-19

RNA viruses, such as SARS-CoV and SARS-CoV-2, are recognised in the body by cytosolic receptors, e.g. RIG-I, and endosomal RNA receptors, e.g. Toll-like receptors (TLRs) [31]. TLRs are expressed by DCs and macrophages [32] while RIG-I receptors are well known for their functions inside macrophages, neutrophils and DCs [33]. Once the viral antigens are recognised, transcription factors such as nuclear factor kappa B (NF-κB) and interferon regulator factor 3 (IRF3) are activated which then translocate into the nucleus of macrophages and DCs and induce the expression of pro-inflammatory cytokines, chemokines and type I IFNs. Activation of type I IFNs leads to a cascade that results in the activation of IFN-stimulated genes and the subsequent expression of antiviral proteins [31]. These antiviral proteins act together to inhibit viral infection, replication and assembly [28].

The first cell type recruited to the site of infection is neutrophils where they are responsible for the recruitment, differentiation and activation of monocytes, DCs and macrophages [34]. Like other viral infections, SARS-CoV-2 has also been shown to recruit these innate immune cells to sites of infection [35]. Neutrophils are also capable of releasing neutrophil extracellular traps which work to immobilise and inactivate extracellular pathogens [29]. SARS-CoV-2 has been shown to stimulate the production of neutrophil extracellular traps, and this was associated with increased levels of reactive oxygen species which are capable of killing pathogens, directly or indirectly [36].

COVID-19 severity is associated with cytokine storm, which is the result of a sharp increase in levels of inflammatory mediators such as interleukin (IL)-2, IL-7, IL-10, G-CSF, IP10, MCP-1, MIP1A and TNFα [37]. Cytokines IL-6 and IL1RA have also been shown to be uniquely elevated in response to COVID-19 in comparison to SARS-CoV-2 negative controls [35]. While the inflammatory response induced by cytokines is important in disease resolution, a cytokine storm induced by an unbalanced response can be harmful [38].

NK cells use receptors such as CD16 and NKG2D to recognise and subsequently kill virus-infected cells by releasing perforins and granzymes, which induce target cell apoptosis and lysis [27]. They also produce IFNγ which aids in mobilising APCs as well as promoting the development of effective antiviral immunity [27]. However, in SARS-CoV-2 infection, a reduction in NK cells has been observed with decreased cytolytic function observed in remaining NK cells [39]. Also, patients not in ICU had significantly higher levels of NK cells compared to those in ICU [40] which is supported by another study suggesting an impairment of immune cytotoxic effector functions [41].

## The adaptive response and COVID-19

Respiratory viruses like SARS-CoV-2 replicate abundantly in the upper respiratory epithelia [42]. APC activation in the respiratory tract and migration into lymph nodes leads to activation of naive CD4^+^, CD8^+^ and memory T cells which are needed to induce the adaptive antiviral response. These activated effector T cells migrate to the site of infection where they mediate the antiviral immune response [43]. CD4^+^ T cells act as helpers by promoting macrophage activation and antibody production as well as aiding CD8^+^ T cells in performing their cytotoxic function efficiently. B cells are capable of recognising and presenting antigens themselves, and once introduced to a foreign antigen, they proliferate and differentiate into antibody-producing plasma cells and memory B cells [27].

A key characteristic of the SARS-CoV-2 infection is lymphopenia which is more pronounced in severe cases [44]. In fact, significantly lower levels of CD8^+^ T cells and increased levels of IL-6 have been shown to be reliable indicators of developing severe COVID-19 [45] while the T cells that remain appear functionally exhausted [46]. Moreover, lymphopenia has also been associated with severe illness and poorer survival in the SARS-CoV-1 viral infection [47] supporting the theory that lymphopenia is negatively correlated with overall survival.

## Unconventional T cells

T cells can be broadly split into two categories: conventional T cells (including CD4^+^ and CD8^+^ T cells) and the lesser studied unconventional or innate-like T cells such as γδ T cells, iNKT cells and MAIT cells, which display features of both the innate and adaptive immune systems [48]. The differing characteristic of these subtypes is the TCR type and diversity as well as the molecules recognised by the respective TCRs. Conventional T cells have a diverse repertoire of αβ-TCRs that recognise classical peptide antigens presented by MHC molecules, while unconventional T cells generally express invariant TCRs that recognise non-peptide antigens in an MHC-unrestricted manner [49].

## γδ T cells

γδ T cells express a TCR composed of a γ-chain bound to a δ-chain [53, 54]. They account for ∼ 0.5–16% of total CD3^+^ cells in various human tissues with a mean of ∼ 4% in peripheral blood [51]. In humans, γδ T cells are grouped according to their TCR δ-chain usage [50] with the main human γδ T cells subsets being Vδ1, Vδ2 and Vδ3 [52]. Human Vδ1 T cells are found primarily in the gut epithelia, dermis, spleen and liver, while Vδ2 cells are found mainly in the peripheral blood and are usually paired with a Vγ9 chain [50]. The less abundant Vδ3 subtype [53] is relatively rare (> 0.5%) in the peripheral blood and is also detectable in mucosal tissues [54] and liver [50]. Vγ9Vδ2 T cells respond to phosphoantigens expressed by microbes [55], while Vδ1 and Vδ3 T cells recognise various phospholipids and glycolipids presented by CD1c [56] and CD1d molecules [57, 61]. Human γδ T cells display broad reactivity to many types of viruses [58].

All human γδ T cell subsets display potent cytotoxic functions and possess cytolytic machinery such as expression of perforin, granzymes and natural cytotoxicity receptors. Ligation of the natural cytotoxicity receptor NKp30 on Vδ1 T cells triggers production of chemokines CCL3, CCL4 and CCL5 which suppress HIV replication [59]. Vδ2 T cells have been shown to mediate killing of influenza-infected target cells through the formation of a functional immune synapse and delivery of perforin to the virally infected cell [60]. The presence of γδ T cells has also been associated with the absence of cytomegalovirus (CMV) in blood while their absence was associated with recurrent CMV presence in kidney transplant patients [61]. In SARS-CoV, the Vδ2 T cell population has been postulated to play a protective role due to the expansion of this cell population 3 months after the onset of disease which was associated with higher anti-SARS-CoV IgG titres [62]. In vitro stimulation of Vδ2 T cells with SARS-CoV showed IFNγ-dependent anti-SARS-CoV activity and an ability to directly kill SARS-CoV-infected cells through their cytotoxic effector function [62].

## γδ T cells in COVID-19

In mild cases of COVID-19, γδ T cell expression was shown to be decreased in peripheral blood [63]. However, a significant increase in CD4^+^ γδ T cells (which usually account for roughly 6–7% of γδ T cells) was observed in response to the infection which was hypothesised to facilitate the activation of adaptive immune cells [63]. Furthermore in mild cases, γδ T cells expressed elevated levels of the costimulatory marker CD25 when compared to healthy donors, but no difference was seen in expression of the early activation marker CD69 [63]. In both mild and severe cases of COVID-19, frequencies and absolute cell numbers of naïve-like (CD45RA^+^CD62L^+^) γδ T cells were increased in blood which was accompanied by a proportional decrease in effector-like (CD45RA^-^CD62L^−^) γδ T cells. It has been hypothesised that effector-like γδ T cells might be recruited to the lungs of COVID-19 patients where they could participate in the immune response against the infection [64].

In severe cases of COVID-19, decreased levels of circulating γδ T cells have been reported [64]. In fact, a gradual reduction in circulating Vδ1 and Vδ2 T cells has been shown to be inversely correlated with disease severity, which is hypothesised to happen due to the activation and infiltration of these cells into the lungs [65]. This study showed that this inverse correlation was more pronounced in Vδ2 cells which suggests a selective activation and infiltration of these cells in the lungs, but this has not yet been proven. Individuals admitted to hospital with COVID-19 have been shown to express significantly lower levels of Vγ9Vδ2 T cells in blood compared with healthy controls [66]. Of 24 patients admitted, six patients (four of whom were in ICU) died while hospitalised and showed lymphocytopenia which included decreased levels of Vγ9Vδ2 T cells. In five surviving patients, the Vδ2 phenotype was monitored and it was observed that 26% of Vγ9Vδ2 T cells shifted to an effector memory cell phenotype over the course of 2 weeks, compared with just 8% of the total T cell population [66]. In addition, CD69 expression was elevated on γδ T cells. However, this was deemed to not be COVID-19 specific but rather a general reflection of a severe inflammatory condition as levels were not significantly changed when compared with non-COVID-19 critically ill controls [67]. Moreover, the blood ratio of immature neutrophils to Vδ2 cells has been shown to be a good prognostic marker for severe COVID, surpassing the prognostic ability of the neutrophil-to-lymphocyte ratio or neutrophil-to-CD8 T cell ratio [65].

## iNKT cells

Natural killer T (NKT) cells can be divided into two subtypes: type I and type II NKT cells. However, type I NKT cells will be the focus of this review as knowledge on type II NKT cells is currently limited. Type I NKT cells are more commonly referred to as invariant NKT (iNKT) cells as their TCR is composed of an invariant α-chain (V-α24/J-α18 in humans) bound to a limited array of β-chains. They represent 0.1–1% of human T cells in the blood and liver but relatively speaking, are one of the more abundant TCR specificities in the body due to their invariant nature [49]. iNKT cells recognise glycolipid antigens presented by CD1d molecules, most notably alpha-galactosylceramide (α-GalCer) [49], but can also be activated by proinflammatory cytokines produced during infection [68].

iNKT cells are important in the regulation of immune responses in autoimmunity, cancer and microbial infection [68]. They are capable of both promoting cell-mediated immunity to tumours and infectious organisms as well as suppressing cell-mediated immunity associated with autoimmune disease and allograft rejection [69]. In the viral setting, iNKT cell numbers have been shown to be decreased in patients with HIV after seroconversion [70]. However, treatment of HIV with α-GalCer, which is a potent iNKT cell stimulant, demonstrated enhanced anti-viral immunity and improved clinical outcomes [71].

## iNKT cells in COVID-19

In moderate cases of COVID-19, an expansion of CD160^+^ NKT cells has been observed which may help to promote quick resolution of the infection through direct cytotoxicity [72]. The *FCGR3A* gene was also shown to be enriched in this cluster which suggests that it may mediate antibody-dependent cell-mediated cytotoxicity. However, the NKT CD160 cluster was notably absent in severe COVID-19 cases [72]. The authors did not specify whether the NKT cell cluster were type I or type II NKT cells and CD160 is expressed by both subtypes [73]. Thus, it is likely that the cluster contained both type I and type II NKT cells.

In severe cases of COVID-19, circulating iNKT cells have been shown to be activated by IL-18 [67] which is a cytokine associated with unconventional T cell activation during viral infections [75–77]. Furthermore, there is an observed decrease in circulating iNKT cells and in iNKT cell production of IFNγ [67]. Of the remaining iNKTs in circulation, levels of those expressing PD-1 and CD69 were increased while high PD-1 expression persisted on iNKT cells from patients in intensive care units at day 15 [67].

## MAIT cells

MAIT cells recognise microbe-derived vitamin B metabolites presented by the major histocompatibility complex class I-related molecule (MR1) and play a putative role in antimicrobial immunity at mucosal sites [77]. MAIT cell TCRs are composed of an invariant α-chain (Vα7.2 in humans) which is paired with either a Vβ2 or Vβ13 chain [78]. MAIT cells account for up to half of all T cells in the liver [79] and 2–5% of T cells in human blood, and while MAIT cell frequencies vary among healthy individuals, low frequencies are commonly associated with disease states [49] such as human T lymphotropic virus type 1 [80], HIV-1 [81] and hepatitis C virus [82]. Although not directly activated by viruses, MAIT cells have been shown to be activated in a TCR-independent manner by cytokines IL-12 and IL-18 [83]. Activated MAIT cells rapidly produce proinflammatory cytokines, including IFNγ, TNFα and IL-17. MAIT cell activation in an IL-18 dependent manner has been demonstrated in RNA virus infections such as dengue virus, hepatitis C and influenza virus [74] and has also been shown to have the potential to promote protective immunity in human influenza infection [84]. In a H1N1 influenza infection model, MAIT cell deficient mice showed greater weight loss and mortality [85]. In vitro, MAIT cells activated by IL-12 and IL-18 have been shown to be capable of suppressing hepatitis C virus replication, which was reversed by the addition of anti-IFNγ [74].

## MAIT cells in COVID-19

During SARS-CoV-2 infection, MAIT cells have been shown to be activated in a receptor-independent manner by IL-18, since high plasma IL-18 levels correlated with CD69 expression on MAIT cells [67]. In both mild and severe infections, decreased frequency of circulating MAIT cells was observed, thought to reflect recruitment of MAIT cells into the airways and possibly activation-induced cell death [71, 87–89]. Indeed, when comparing endotracheal aspirates to matched patient blood samples, a higher frequency of MAIT cells was observed in the airways (0.4% in blood vs 3.36% in aspirates in COVID-19 patients while healthy donors had 3.5% MAIT cell frequency in blood) [67]. Moreover, MAIT cells have been shown to be enriched within T cells infiltrating the airways of COVID-19 patients, which is consistent with the decline in circulating MAIT cells [86, 87]. An inverse correlation between MAIT cell frequency and plasma levels of IL-17C was also shown [86]. IL-17C is a proinflammatory cytokine preferentially produced in lung, skin and colon epithelial cells and is involved in both host defence and inflammatory diseases of such tissues [89]. This finding supports the possible link between MAIT cell recruitment and lung epithelial inflammation observed in the SARS-CoV-2 infection [86].

MAIT cells show signs of activation during the SARS-CoV-2 infection, though how this links with clinical outcome is unclear. In both mild and severe cases of COVID-19, increased expression of the early activation marker CD69 on MAIT cells and diminished expression of the homing receptor chemokine C-X-C motif receptor 3 (CXCR3) has been reported [71, 87, 88, 90]. MAIT cells usually express CXCR3 which is involved in lung homing [90]. A positive clinical correlation was found between CD69 expression by MAIT and NKT cells in COVID-19 patients at time of admission and reduced hypoxemia after 7 days [67].

The link between MAIT cell activation and COVID-19 outcomes was further interrogated using Phenograph clustering [86], a graph-based method for identifying subpopulations in high-dimensional single-cell data [91]. Four Phenograph MAIT cell clusters were found to be overrepresented in patients who died of COVID-19 and these clusters were all characterised by high CD69 expression and low/very low levels of CXCR3 [86]. In this study, the COVID-19 patients who had died were shown to have had higher CD69 expression than those who survived but retrospective sampling of a biobank group of severely ill patients saw no association between CD69 and outcome. However, when comparing the deceased and retrospective groups, it was found that the retrospective group was sampled 25 days after symptom onset while the deceased patients had been sampled 14 days after symptom onset. This study observed that samples obtained closer to when symptoms first manifested showed higher CD69 expression by MAIT cells, but the expression levels decreased when samples were obtained later after the onset of symptoms. As a result of this, it was postulated that high levels of MAIT activation early in disease may be associated with poorer outcome [86]. CD69 upregulation and CXCR3 downregulation patterns were more pronounced in MAIT cells compared to conventional CD4^+^ and CD8^+^ T cells, and CD69 expression was inversely linked with CXCR3 expression on MAIT cells [86]. This circulating CD69^+^CXCR3^-^ MAIT cell phenotype was reproduced in the airways where MAIT cells were also shown to be the main source of IL-17A [86]. Significantly higher levels of IL-17A have been observed in both mild and severe cases of COVID-19, with highest expression observed in severe cases [92]. The authors also demonstrated that, while IL-17A is not considered a good marker for disease severity, it is capable of activating inflammatory pathways that may result in tissue damage and exacerbation of disease, meaning it likely plays a deleterious role in COVID-19 pathogenesis.

In a cohort of 43 COVID-19 patients and 25 healthy controls, circulating MAIT cells displayed reduced frequency and an activated phenotype in individuals with COVID-19, regardless of disease severity, as characterised by high expression of IL-17A, TNFα, CD38, CD69 and HLA-DR [88]. Elevated expression of the activation marker HLA-DR by MAIT cells correlated with disease severity, as measured by the Simplified Acute Physiology Score (SAPS) II score, which estimates the probability of mortality for ICU patients. This study did not note any significant changes in the frequency of conventional T cells compared to healthy controls. However, MAIT and Vδ2 T cells were significantly reduced in peripheral blood from COVID-19 patients and levels remained low in convalescent patients when compared to acute cases. CD8 expression by MAIT cells was markedly lower in individuals with COVID-19. In vitro stimulation assays revealed an altered cytokine production profile and impaired cytotoxic function [88]. When MAIT cells were stimulated with *E. coli*, typical upregulation of IFNγ was observed but levels of TNFα and IL-17A were significantly lower in COVID-19-positive individuals compared to healthy controls. Ex vivo baseline levels of granzyme B and perforin were high in MAIT cells from the COVID-19 cohort compared to controls, and upregulation was not observed in response to stimulation with *E. coli*. When stimulated with cytokines IL-12 and IL-18, impaired upregulation of IL-17A, TNFα, granzyme B and perforin was observed in the COVID-19 cohort, and a reduction in perforin was observed.

MAIT cells have been implicated in stimulating antiviral T cell effector responses after vaccination with a leading SARS-CoV-2 candidate vaccine, ChAdOx1, the AstraZeneca/Oxford University nCoV-19 vaccine [93]. ChAdOx1 vaccination drove MAIT cell activation via IFNα, TNF and IL-18, and MAIT cell activation positively correlated with vaccine-induced T cell responses in both human and mouse models. Activation was shown to be dose-dependent and resulted in upregulation of CD69, granzyme B and IFNγ production by MAIT cells. MAIT cell activating cytokines were shown to emanate from viral vector transduced cells, namely IFNα from plasmacytoid DCs, and IL-18 and TNF from monocytes. The mechanism by which MAIT cells activate CD8 T cells remains undefined, although the authors suggest that the chemokine CXCL10 may be a candidate signalling molecule. The immunogenicity of the adenoviral vector was tightly linked to MAIT cell activation, placing MAIT cells as an important intermediate bridging innate and adaptive immunity.

Taken together, the data shows that MAIT cell frequency is reduced in the periphery and increased in the lung in COVID-19 patients, where an early activated phenotype is associated with poorer clinical outcome, signifying a pathogenic role for MAIT cells [71, 87–89]. However, MAIT cells have also been shown to boost CD8 T cell effector responses to adenoviral vaccines and their activation is linked to vaccine immunogenicity [93]. Further research is required to dissect these differing contributions to antiviral immunity in the COVID-19 setting, but it appears likely that MAIT cell effector responses may have different impacts depending on disease stage.

## Therapeutic potential of unconventional T cells in COVID-19

Unconventional T cells have been under investigation as immunotherapeutic agents in the last few decades, mainly in the area of cancer due to their potent cytotoxic ability, and have proven to be well tolerated and safe as cell-based treatments [94]. Immunotherapies utilising unconventional T cells could potentially extend to COVID-19 also. One such interventional clinical trial is already in phase 1 and aims to determine the safety and efficacy of an unmodified, allogenic iNKT cell therapy named agenT-797 in moderate-to-severe COVID-19 patients with ARDS, requiring mechanical ventilation (NCT no. NCT04582201) [95]. This trial aims to determine whether iNKT cell therapy has the potential to clear the virus, decrease inflammation and prevent reinfection.

Another proposed novel approach to COVID-19 treatment is boosting Vδ2 T cell antiviral activity through administration of bisphosphonate-based therapy [96]. Zoledronic acid is an amino-bisphosphonate drug that drives expansion of Vδ2 T cells, and Vδ2 T cells inhibit SARS-CoV-2 virion release by inhibiting small GTPases in the endosomal pathway [97]. Vδ2 T cells have already demonstrated prognostic importance, with immature-neutrophil-to-Vδ2 ratio shown to be a superior blood prognostic marker for predicting COVID-19 severity, when compared to the neutrophil-to-lymphocyte ratio and the neutrophil-to-CD8 T cell ratio [65]. Therefore, elucidating the role of unconventional T cells in COVID-19 may reveal novel therapies that could aid in COVID-related recovery. Their potential as therapeutic agents is strengthened by the fact that Vδ2 cells have been shown to kill virus-infected cells in an IFNγ-dependent manner in SARS-CoV infection [62]. However, further research is required to determine whether this also occurs in SARS-CoV-2 infection and data describing unconventional T cell effector functions in this setting is currently lacking.

MAIT cells have been shown to be recruited into the airways during SARS-CoV-2 infection and their activation is linked with worse clinical outcomes in COVID-19 [87, 88]. One potential driving factor is that MAIT cells are a source of IL-17 in severe COVID-19 [86]. Therapeutic targeting of IL-17 is hypothesised to inhibit development of ARDS [98]. It is possible that MAIT cell functions could also be therapeutically inhibited using inhibitory riboflavin biosynthesis pathway derivatives [99]. However, given the observed importance of MAIT cell activation in promoting effector T cell vaccine responses and promoting vaccine immunogenicity, it may not be advisable to completely inhibit MAIT cell activation in this setting. Further work must be done to determine MAIT cell functions at different disease stages in order to optimise clinical outcomes.

## Conclusion

SARS-CoV-2 infection triggers an inflammatory response which can become severe and result in cytokine storm and serious debilitating illness. Emerging investigations into understudied unconventional T cell subsets in COVID-19 reveal intriguing information implicating their involvement in both pathogenesis and resolution of COVID-19, though the underlying mechanisms and stage-dependent functions are as yet undefined. Unconventional T cell subsets have been shown to become activated in response to viral infections including COVID-19 and show evidence of protective antiviral responses but whether such effector functions can be successfully harnessed for therapy requires further study. It is likely that unconventional T cells become activated in response to cellular or inflammatory stimuli rather than through direct viral recognition. This raises the possibility for targeted therapeutic manipulation of these cells using exogenous microbe-derived, metabolite- or cytokine-based stimuli. In addition to enhancing understanding of unconventional T cells in primary SARS-CoV-2 infection, loss or dysfunction of such cells may also increase susceptibility to other microbial infections, driving secondary infections which can lead to serious complications such as sepsis. There is therefore a need to learn more about the complex antimicrobial effector functions of unconventional T cell subsets, cells which bridge innate and adaptive immunity.
